# Downregulation of 14q32 microRNAs in Primary Human Desmoplastic Medulloblastoma

**DOI:** 10.3389/fonc.2013.00254

**Published:** 2013-09-25

**Authors:** Danielle Ribeiro Lucon, Cristiane de Souza Rocha, Rogerio Bastos Craveiro, Dagmar Dilloo, Izilda A. Cardinalli, Denise Pontes Cavalcanti, Simone dos Santos Aguiar, Claudia Maurer-Morelli, Jose Andres Yunes

**Affiliations:** ^1^Centro Infantil Boldrini, Campinas, Brazil; ^2^Departamento de Genética Médica, Faculdade de Ciências Médicas, Universidade Estadual de Campinas, Campinas, Brazil; ^3^University Children’s Hospital Bonn, Department of Pediatric Hematology and Oncology, Bonn, Germany

**Keywords:** 14q32 miRNA cluster, desmoplastic medulloblastoma, *ESRRG*, miR-129-5p, miRNA profile

## Abstract

Medulloblastoma (MB) is one of the most common pediatric cancers, likely originating from abnormal development of cerebellar progenitor neurons. MicroRNA (miRNA) has been shown to play an important role in the development of the central nervous system. Microarray analysis was used to investigate miRNA expression in desmoplastic MB from patients diagnosed at a young age (1 or 2 years old). Normal fetal or newborn cerebellum was used as control. A total of 84 differentially expressed miRNAs (64 downregulated and 20 upregulated) were found. Most downregulated miRNAs (32/64) were found to belong to the cluster of miRNAs at the 14q32 locus, suggesting that this miRNA locus is regulated as a module in MB. Possible mechanisms of 14q32 miRNAs downregulation were investigated by the analysis of publicly available gene expression data sets. First, expression of estrogen-related receptor-γ (*ESRRG*), a reported positive transcriptional regulator of some 14q32 miRNAs, was found downregulated in desmoplastic MB. Second, expression of the parentally imprinted gene *MEG3* was lower in MB in comparison to normal cerebellum, suggesting a possible epigenetic silencing of the 14q32 locus. miR-129-5p (11p11.2/7q32.1), miR-206 (6p12.2), and miR-323-3p (14q32.2), were chosen for functional studies in DAOY cells. Overexpression of miR-129-5p using mimics decreased DAOY proliferation. No effect was found with miR-206 or miR-323 mimics.

## Introduction

Medulloblastoma (MB) is an embryonic tumor of the cerebellum and the most common malignant brain tumor in childhood, likely originating from abnormal development of cerebellar progenitor neurons (1, 2). Transcriptional profiling of large number of MB samples unraveled the existence of at least four distinct molecular subgroups: (i) Wingless (Wnt) group, (ii) Sonic Hedgehog (SHH) group, (iii) Group 3 and (iv) Group 4 (3, 4). These various subtypes of MB are suggested to arise from different populations of precursor or stem cells which form the cerebellum (5–8). This transcriptome-based classification has opened new avenues for the understanding of the molecular mechanism contributing to MB.

MicroRNAs (miRNAs) are suggested to play an important role in controlling the development of the central nervous system (CNS) by regulating cell proliferation and differentiation, as well as apoptosis (9). miRNAs are small non-coding RNA molecules of ∼22–25 nucleotides that post-transcriptionally downregulate gene expression by binding the 3′-untranslated region (UTR) of protein coding transcripts, resulting in either mRNA cleavage or translational repression (10, 11). miRNA expression profiling of both mouse and human MB has led to the identification of signatures associated with the molecular subgroups of MB, tumor diagnosis, and response to treatment, as well as novel targets of potential clinical relevance (12–15). Previous studies, however, interrogated limited number of miRNAs and included adult cerebellum in the normal control group. We investigated the expression profile of 847 miRNA in primary human desmoplastic MB of younger children in comparison to normal fetuses or newborn cerebellum. Eighty-four miRNAs were found to be differential expressed in MB, most of them belonging to the cluster 14q32. Possible mechanisms of 14q32 locus downregulation were investigated by the analysis of publicly available gene expression data set. Functional studies using mimic miR-129-5p (11p11.2/7q32.1), miR-206 (6p12.2), and miR-323-3p (14q32.2) and the DAOY cell line, suggested a suppressive role for miR-129-5p in MB proliferation.

## Materials and Methods

### Primary medulloblastoma tissue samples

**Figure A1:**
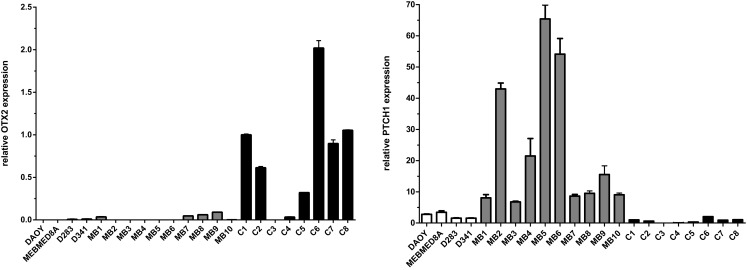
**Expression of OTX2 and PTCH1 in the different primary medulloblastomas (MB), medulloblastoma cell lines (named), and normal cerebellum (C)**. Total RNA (100 ng) was reverse transcribed and gene expression was measured by qPCR, in triplicates, using the SYBR method. Primers are available upon request to the authors. Values were normalized to the HPRT endogenous control gene. Relative expression values were calculated using the 2^−ΔΔ*Ct*^ method.

Surgical specimens were obtained from 1 to 5 years old children (*n* = 10), with desmoplastic MB (Table 1). Of note, microarray analyses were performed with MB samples from children with 1–2 years old. Desmoplastic MBs belong, with rare exceptions, to the SHH molecular subgroup (13–16). All MB samples used in the present study had high mRNA levels of *PTCH1* and low levels of *OTX2* (Figure A1 in Appendix), in comparison to normal cerebellum, which is in keeping with the differential transcriptional profile of SHH tumors (3). Normal cerebellum tissues were obtained from 22 to 39 weeks old fetal and newborn (NW) autopsy (*n* = 8) (Table 2). Ethical approvals were obtained from the Ethical Research Committee of the Faculdade de Ciências Médicas (n°656/2009), CAISM (n°064/2010), the Ethical Research Committee of Centro Infantil Boldrini (n°1.90-030710), and National Committee of Ethics in Research (CONEP) n°0005.0.144.146-09. Subtyping of MB was obtained by histological analysis.

**Table 1:** **Summary of the medulloblastoma samples included in the study**.

Medulloblastoma	Age at diagnosis	Gender	Histology
MB 1^a^	1	M	N/D
MB 2^a^	1	F	N/D
MB 3^a^	2	M	D
MB 4^a^	2	M	N/D
MB 5^a^	1	M	N/D
MB 6^a^	2	M	N/D
MB 7	5	M	N/D
MB 8	5	M	D
MB 9	4	M	D
MB 10	5	M	N/D

^a^Samples used in Affymetrix miRNA microarray analysis; F = female; M = male; D = desmoplastic; N/D = nodular/desmoplastic.

**Table 2:** **Summary of the normal cerebellum tissues**.

Normal cerebellum	Gestational age	Gender	Diagnosis
C1	37	M	Bilateral renal agenesis
C2^a^	39	M	Hydropsy
C3^a^	22	–	NM
C4^a^	31	M	NM
C5^a^	36	–	NM
C6^a^	24	M	NM
C7	30	–	Cardiopathy
C8^a^	26	M	NM

^a^Samples used in Affymetrix miRNA microarray analysis; F = female; M = male; NM = no malformation and no aneuploidy.

### Total RNA isolation and analysis of global miRNA expression

Total RNA was extracted by Trizol™ (Invitrogen, Carlsbad, CA, USA) according to the manufacturer’s instructions, with an additional overnight precipitation step at −20°C with isopropanol (Merck). RNA quantification was carried out in a *Qubit Quantitation Platform* (Invitrogen) and RNA quality was analyzed via gel electrophoresis. Five hundred nanograms of RNA from 12 samples (6 MB and 6 fetal cerebellum) were labeled with the 3′-DNA FlashTag Biotin HSR kit (Genisphere, Hatfield, PA, USA) and hybridized to GeneChip miRNA Array 1.0 (Affymetrix Inc., Santa Clara, CA, USA), which comprises 847 human miRNAs. Data was acquired using a GeneChip Scanner 3000 7G (Affymetrix).

### Validation of miRNA deregulation by quantitative real-time PCR

Reverse transcription (RT) and quantitative real-time RT-PCR (RT-qPCR) analysis were carried out using commercially available TaqMan microRNA assays (Applied Biosystems, Foster City, CA, USA) and a 7500 Real-time PCR System (Applied Biosystems). RT reactions (50 ng of total RNA) were performed in a 15 μl final volume containing specific stem-loop primers for each miRNA (129-5p, 206, 323-3p, 495, and internal control small RNA, RNU6B), 10× RT Buffer, dNTPs, reverse transcriptase, RNase inhibitor, and water in 96-well plates. Thermal cycling included 30 min at 16°C, 30 min at 42°C, and a final step of RT inactivation for 5 min at 85°C. PCR reactions were performed in a 10 μl final volume containing 5 μl TaqMan Universal Master Mix II, without UNG (Applied Biosystems), 3.5 μl water, 0.5 μl TaqMan microRNA assay, and 1 μl cDNA. Thermal cycling included an initial step of 10 min at 95°C for Taq activation followed by 40 cycles of 15 s denaturation at 95°C and 1 min of annealing/extension at 60°C. Each reaction was performed in triplicate and the miRNAs expression levels were normalized against RNU6B. The threshold cycle numbers (*C*t) were calculated by relative quantification using the 2^−ΔΔ*Ct*^ method, as described by Livak and Schmittgen (17). One of the control samples was chosen as calibrator.

### Cell lines

Four human MB cell lines were utilized: DAOY (HTB 186), D283 Med (HTB185), and D431 Med (HTB-187) were obtained from American Type Culture Collection (ATCC). The MB cell line, MEB-Med-8A, was kindly provided by Prof. T. Pietsch (18). The MB cell lines DAOY, D283 Med, and MEB-Med-8A were maintained in High Glucose Dulbecco’s Modified Eagle Medium (DMEM) supplemented with 1 mM sodium pyruvate (PAA), l-glutamine, 1% penicillin/streptomycin (Invitrogen, Karlsruhe, Germany), and 10% fetal bovine serum (FBS, Invitrogen). The MB cell line D341 Med was maintained in DMEM with l-glutamine supplemented with 1 mM sodium pyruvate, 1% penicillin/streptomycin, and 10% Human Serum (HS, PAA, UK).

### Transient transfection of miRNAs

DAOY cells (1.5 × 10^5^) were seeded in six-well plates in 2 ml of RPMI-1640 medium (Cultilab, Campinas, Brazil) supplemented with 10% FBS (Sigma-Aldrich, St. Louis, MO, USA) and penicillin/streptavidin (Cultilab). Transfection of miRVana miRNA mimics (Invitrogen Ambion, Austin, TX, USA) of miR-206, miR-129-5p, miR-323-3p, or miRVana miRNA mimic negative control #1 (referred to as scrambled) was carried out 24 h after seeding, in a final concentration of 3 nM, using Lipofectamine RNAiMAX reagent (Invitrogen) according to the manufacturer’s recommendation. Efficiency of transfection was evaluated 24 post-transfection by RT-qPCR using total RNA.

### Cell viability: MTS assay

Cell survival/proliferation after the transfection with mimic-miRNAs was evaluated by using the CellTiter 96 AQueous One Solution Cell Proliferation Assay (Promega, Wallisellen, Switzerland), a colorimetric [3-(4,5-dimethylthiazol-2-yl)-5-(3-carboxymethoxyphenyl)-2-(4-sulfophenyl)]-2H-tetrazolium inner salt (MTS) assay. Briefly, mimic-miR-206, mimic-miR-129-5p, and mimic-miR-323-3p or mimic-negative control #1 transfected cells were harvested 20 h after transfection and seeded in triplicate in 96-well plate (1,500 cells/well) in serum-free RPMI-1640 (Cultilab). At 24, 48, or 72 h post-transfection (i.e., 4, 28, or 52 h after passage to the 96-well plate) cells were incubated for 1 h with MTS reagent and absorbance read at 492 nm (reference wavelength 620 nm) using an ASYS Expert Plus Microplate Reader (Biochrom, Holliston, MA, USA). Three independent experiments were performed.

### Apoptosis assay

DAOY cells transfected with miR-206, 129-5p, 323-3p, or scramble mimics were cultured for 24 h in serum-free RPMI-1640 (Cultilab), harvested and part of it was resuspended in the appropriate binding buffer, stained with FITC-conjugated Annexin V (BD Biosciences, San Jose, CA, USA) and propidium iodide at room temperature for 15 min, and subsequently analyzed by flow cytometry in a FACS Canto II (Becton Dickinson). The remaining cells were replated in six-well plates for another 24 h culture period in serum-free RPMI-1640 (Cultilab) and harvested 48 h post-transfection for Annexin V labeling.

### Statistical analysis and bioinformatics methods to signaling pathway prediction

MicroRNA expression was analyzed in R environment^1^ using the packages Affy and RankProd from Bioconductor (19– 21). The MB miRNA profile was compared to the cerebellum profile. Differentially expressed miRNAs were selected according to the fold change ≥2.00 and *p*-value ≤0.05. Heat maps were created using tools of the MetaboAnalyst 2.0^2^. Signaling pathways were prospected by DIANA-miRPath (microT-v4.0, beta version)^3^. The input dataset enrichment analysis was performed by Pearson’s chi-squared test and each pathway was represented by the negative natural logarithm of the *P*-value (−ln *P*). The Ingenuity Pathway Analysis (IPA) software^4^ was used to identify possible pathways associated to differentially expressed miRNAs.

Comparisons of RT-qPCR values from MB versus normal cerebellum were performed by the Mann–Whitney test. Cell proliferation results, at each time point, from mimic miRNA transfections versus mimic-negative control #1 were analyzed by the two-tailed unpaired *t*-test. Alpha error of *P* = 0.05 was tolerated. The GraphPad Prism 5 software was used throughout.

## Results

### Identification of differentially expressed miRNAs in desmoplastic MBs of 1–2 years old children

**Figure 1:**
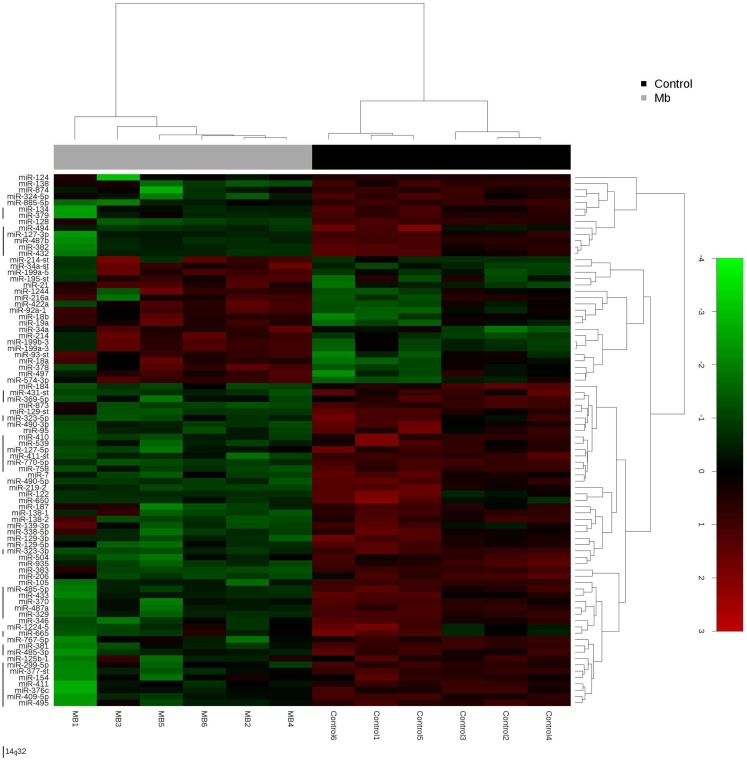
**Hierarchical clustering analysis of medulloblastoma and normal cerebellum**. Unsupervised hierarchical cluster analysis representing the 84 miRNAs expressed in medulloblastoma samples versus normal cerebellum. 14q32 miRNAs are depicted by a left vertical line. -st, star.

Global miRNA profiles were generated for primary MB of the desmoplastic subtype and most likely SHH molecular subgroup (*n* = 6), and normal fetal/NW cerebellum (*n* = 6). Eighty-four miRNAs (64 miRNAs downregulated and 20 miRNAs upregulated) were considered to be differentially expressed (fold change ≥2.0, *p* ≤ 0.05) in MB in comparison to normal fetal/NW cerebellum (Table 3; Figure 1). Among these 84 miRNAs, 46 had been previously described as deregulated in human primary MB (Table 3), and only 8 were previously validated by functional assays (Table 4). Upregulation of miRNAs from the miR-17 ∼ 92 cluster (in this work miR-18a, 19a, and 92a-1) and downregulation of miR-324-5p were previously described in human MB of the SHH subgroup (12, 13). Of especial note, 32 of the 64 downregulated miRNAs belong to a large cluster on human chromosome 14q32 (Figure 1; Table 3).

**Table 3:** **Deregulated miRNA in desmoplastic medulloblastoma compared to normal cerebellum**.

Our miRNA profile (84)	Chromosomal localization	Fold change	Reference
**DOWNREGULATED**			
hsa-miR-206	6p12.2	−7.53	(29)
hsa-miR-219-2-3p	9q33.3	−6.64	(52)
hsa-miR-383	8p22	−6.56	(12, 55, 56)
hsa-miR-138	16q13.3/3p21.32	−5.16	(12, 14)
hsa-miR-323-3p	14q32.2	−4.96	(12, 52)
hsa-miR-122	18q21.31	−4.82	
hsa-miR-105	Xq28	−4.66	
hsa-miR-129-5p	11p11.2/7q32.1	−4.56	(23)
hsa-miR-935	19q13.43	−4.53	(52)
hsa-miR-329	14q32.2	−4.48	
hsa-miR-129-3p	11p11.2/7q32.1	−4.43	
hsa-miR-650	22q11.21	−4.19	
hsa-miR-184	15q24.3	−4.14	
hsa-miR-370	14q32.2	−3.99	(12)
hsa-miR-433	14q32.2	−3.96	(29)
hsa-miR-138-2*	16q13.3/3p21.32	−3.91	
hsa-miR-487b	14q32.2	−3.82	(29)
hsa-miR-487a	14q32.2	−3.78	
hsa-miR-758	14q32.2	−3.65	
hsa-miR-485-5p	14q32.2	−3.60	
hsa-miR-138-1*	16q13.3/3p21.32	−3.55	
hsa-miR-382	14q32.2	−3.53	(12, 29)
hsa-miR-504	Xq26.3	−3.45	(52)
hsa-miR-128	2q21.3/3p22.3	−3.43	(12, 14, 51, 59)
hsa-miR-490-5p	7q33	−3.42	
hsa-miR-770-5p	14q32.2	−3.35	
hsa-miR-410	14q32.2	−3.30	(29)
hsa-miR-432	14q32.2	−3.29	
hsa-miR-485-3p	14q32.2	−3.02	
hsa-miR-490-3p	7q33	−2.88	
hsa-miR-381	14q32.2	−2.73	(12)
hsa-miR-377*	14q32.2	−2.72	
hsa-miR-7	15q25.3/19p13.3/9q21.32	−2.72	(12, 14)
hsa-miR-124	20p23.1/8q12.3/8p23.1	−2.71	(12, 14, 29, 48, 49)
hsa-miR-323-5p	14q32.31	−2.69	(12)
hsa-miR-873	9p21.1	−2.65	
hsa-miR-129*	11p11.2/7q32.1	−2.63	
hsa-miR-338-5p	17q25.3	−2.61	(14)
hsa-miR-409-5p	14q32.2	−2.61	
hsa-miR-874	5q31.2	−2.46	
hsa-miR-495	14q32.2	−2.46	(52)
hsa-miR-885-5p	3p25.3	−2.45	
hsa-miR-376c	14q32.2	−2.43	(52)
hsa-miR-299-5p	14q32.2	−2.41	
hsa-miR-539	14q32.2	−2.40	(52)
hsa-miR-127-5p	14q32.2	−2.35	(12, 29)
hsa-miR-127-3p	14q32.2	−2.35	(52, 59)
hsa-miR-411*	14q32.2	−2.30	
hsa-miR-125b-1*	11q24.1/21q21.1	−2.27	
hsa-miR-411	14q32.2	−2.23	(29)
hsa-miR-379	14q32.2	−2.22	(29, 52)
hsa-miR-431*	14q32.2	−2.22	
hsa-miR-767-5p	Xq28	−2.20	
hsa-miR-139-3p	11q13.4	−2.17	
hsa-miR-154	14q32.2	−2.16	(12)
hsa-miR-1224-5p	3q27.2	−2.15	
hsa-miR-187	18q12.1	−2.14	(12)
hsa-miR-95	4p16.1	−2.10	(14)
hsa-miR-369-5p	14q32.2	−2.05	
hsa-miR-665	14q32.2	−2.05	
hsa-miR-494	14q32.2	−2.03	(52)
hsa-miR-134	14q32.2	−2.03	(12, 29)
hsa-miR-346	10q23.2	−2.01	(12, 13)
hsa-miR-324-5p	17p13.1	−2.00	(12, 50)
**UPREGULATED**			
hsa-miR-199b-3p	9q33.3	4.56	(12)
hsa-miR-199a-3p	19p13.2/1q24.1	4.49	
hsa-miR-199a-5p	19p13.2/1q24.1	4.14	(28)
hsa-miR-21	17q22	3.70	(12–14, 29, 31)
hsa-miR-214	1q24.2	3.59	
hsa-miR-19a	13q31.3	3.11	(12–14)
hsa-miR-92a-1*	13q31.3/Xq26.2	3.06	
hsa-miR-214*	1q24.2	2.93	
hsa-miR-34a	1p36.23	2.78	(13, 30, 53)
hsa-miR-18b	Xq26.2	2.74	
hsa-miR-422a	15q22.2	2.72	(14)
hsa-miR-34a*	1p36.23	2.58	(14)
hsa-miR-574-3p	4p14	2.49	(14)
hsa-miR-378	5q32	2.39	(14)
hsa-miR-1244	12p13.2/12p13.31/2q37.1/5q23.1	2.39	
hsa-miR-18a	13q31.3	2.39	(12–14)
hsa-miR-93*	7q22.1	2.26	
hsa-miR-497	17p13.1	2.17	(13)
hsa-miR-195*	17p13.1	2.14	
hsa-miR-216a	2p16.1	2.07	(14)

Deregulated miRNAs previously described in human primary medulloblastoma compared with normal cerebellum or cell lines: (i) miRNAs found downregulated in Ref. (12–14, 23, 26, 29, 48–52, 55, 56, 59); (ii) miRNAs found upregulated in Ref. (12–14, 28–31, 53).

**Table 4:** **miRNAs validated by functional assay in human and mouse medulloblastoma**.

miRNA	Deregulation	Cells	Functional assay^a^	Target genes	Reference
124	Down	Primary human MB, cell lines	↑Cell cycle progression at G1	CDK6	(48, 49)
			↓Cell proliferation	SLC16A1	
324-5p	Down	Primary human and mouse MB, cell lines	↓Cell proliferation	SMO	(50)
326				GLI1	
125p	
9	Down	Primary human MB, cell lines	↑Apoptosis	Trkc	(12)
125a			↓Cell proliferation	
199b-5p	Up	Primary human and mouse MB, cell lines	↑Cell cycle progression at G1	HES1	(28)
			↓Cell proliferation	
128	Down	Primary human and mouse MB	↓Cell proliferation	BMI-1	(51)
			↑Cell senescence	
21	Up	Primary human MB, cell lines	↓Cell migration	PDCD4	(31)
935	Down	Primary human MB, cell lines	–	KIAA0232	(52 )
				SLC5A3	
				TBC1D9	
				ZFAND6	
34a	Down	MB cell lines	↑Apoptosis	MAGE-A	(30, 53)
			↑Cell cycle progression at S/phase and G2/M	Dll1	
			↓Cell proliferation	Notch1	
			↑Cell senescence	Notch2	
512-5p	Down	Primary human MB, cell lines	–	MYCC	(54 )
383	Down	Primary human MB, cell lines	↑Apoptosis	PRDX3	(55, 56)
			↑Cell cycle progression at G1	
			↓Cell proliferation	
183 ∼96 ∼	Down	MB cell lines	↑Cell cycle progression at	AKT	(57)
182			G0/G1 and G2		
			↓Cell migration	
			↓Cell proliferation	
218	Down	MB cell lines	↓Cell migration	CDK6	(58)
			↓Cell proliferation	REST	

Underlined miRNAs are miRNAs also found deregulated in the present study;

^a^results of ectopic expression or knockdown assays; MB = medulloblastoma.

### Signaling pathways analysis by DIANA

Signaling pathways putatively altered by MB deregulated miRNA were depicted by DIANA-miRPath. The list of the top 20 pathways is shown in Table 5. The Ribosome pathway was only pointed by the list of downregulated miRNAs. Adherens junction, oxidative phosphorylation, and TGF-beta signaling pathways showed higher enrichment when the list of downregulated miRNAs was used in the analysis. On the other hand, the MAPK pathway and genes associated to cancer showed higher enrichment when upregulated miRNAs were used in the analysis.

**Table 5:** **Top 20 pathways predicted by DIANA-miRPath analysis**.

Pathway signaling	All deregulated miRNAs	Downregulated miRNAs	Upregulated miRNAs	14q32 miRNAs
*P*-value^a^
Ribosome	30.03	25.34	–	17.78
Axon guidance	24.96	19.98	17.95	17.7
Wnt signaling pathway	18.6	19.34	17.71	16.99
Focal adhesion	16.65	17.02	15.13	14.73
Adherens junction	16.23	20.37	12.18	16.6
Oxidative phosphorylation	14.95	14.76	7.34	14.45
ErbB signaling pathway	14.8	11.03	9.14	9.02
Metabolism of xenobiotics by cytochrome P450	14.06	15.33	3.15	10.01
Renal cell carcinoma	13.48	13.21	9.35	11.64
TGF-beta signaling pathway	12.4	14.11	5.64	17.56
Regulation of actin cytoskeleton	12.25	11.52	7.86	10.63
Chronic myeloid leukemia	12.06	11.02	8.14	10.54
MAPK signaling pathway	11.86	9.81	17.06	12.4
Colorectal cancer	11.72	13.24	9.79	16.92
Glioma	10	8.47	16.21	7.23
Pancreatic cancer	9.69	9.61	13.18	5.83
Melanogenesis	9.4	9.32	9.07	10.59
Ubiquitin mediated proteolysis	9.28	10.09	6.63	11.11
Prostate cancer	9.1	7	18.2	6.06
Insulin signaling pathway	9	8.54	5.86	4.65

^a^The negative natural logarithm of the enrichment *P*-value calculated for the specific pathway. Underlined shows higher enrichment in downregulated, upregulated, or 14q32 miRNAs lists.

Interestingly, oxidative phosphorylation, TGF-beta signaling pathway, and ubiquitin mediated proteolysis were enriched in the list of 14q32 miRNAs.

### Ingenuity pathway analysis

Network analysis by IPA identified two networks as putative targets for 73 out of the 84 MB miRNAs. Networks were prospected considering only relationships that were experimentally observed. Interestingly, both networks were enriched with miRNAs belonging to the 14q32 cluster.

**Figure 2:**
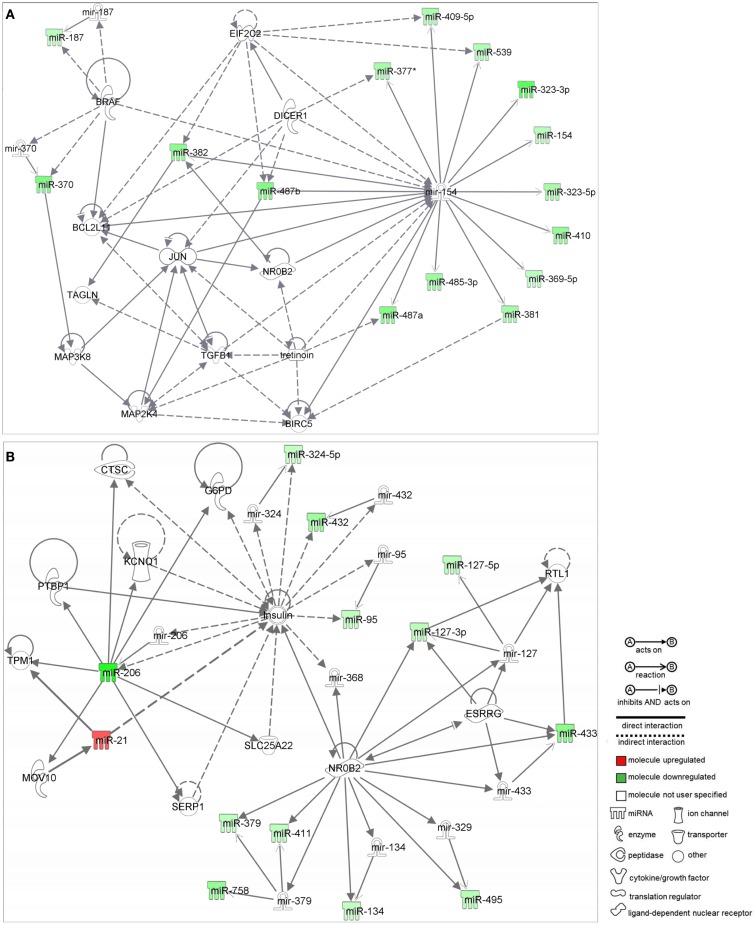
**Ingenuity pathway analysis networks (IPA)**. **(A)** Network 1; **(B)** Network 2; both were constructed with deregulated miRNAs in medulloblastoma.

Network 1 (Figure 2A) included 13 miRNAs of the 14q32 cluster (also known as miR-154 cluster), which were all downregulated in MB samples (miR-154, 323-3p, 323-5p, 369-5p, 377*, 381, 382, 409-5p, 410, 485-3p, 487a, 487b, 539) and were depicted by IPA as having direct interactions with *BCL2L11*, *JUN*, *BIRC5*, *MAP2K4*, and *NR0B2*. *BIRC5* and *BCL-2* have anti-apoptotic roles, and are expected to be at increasing levels in MB as all miRNAs connecting to these genes were found downregulated (Figure 2A). *NR0B2* and *JUN* were suggested in this network as candidate genes controlling the expression of the 14q32 miRNA cluster.

Again, most miRNAs shown in Network 2 (Figure 2B) belong to the 14q32 cluster (miR-127-3p, 127-5p, 134, 379, 411, 432, 433, 495, and 758). In this case, *NR0B2* and estrogen-related receptor-γ (*ESRRG*) were suggested as candidate genes controlling the expression of the 14q32 miRNA cluster. Insulin appeared in Network 2 as indirectly controlling the expression of miR-206, 324-5p, 432, and 95.

### RT-qPCR validation of some deregulated miRNAs

miR-323-3p and 495, both belonging to cluster 14q32, were chosen for validation by RT-qPCR. In addition, miR-206 and miR-129-5p were chosen for analysis because of their high fold change (see Table 3), lack of previous functional studies and possible oncogenic role. miR-206 expression was reported to inhibit cell proliferation in breast cancer cells (22). miR-129 is reported to be significantly downregulated in pediatric brain tumors compared to normal tissues (23). Most importantly, miR-129 downregulation is associated to SOX4 overexpression in endometrial and gastric cancers (24, 25). SOX4 is upregulated and has prognostic impact in MB (26, 27).

**Figure 3:**
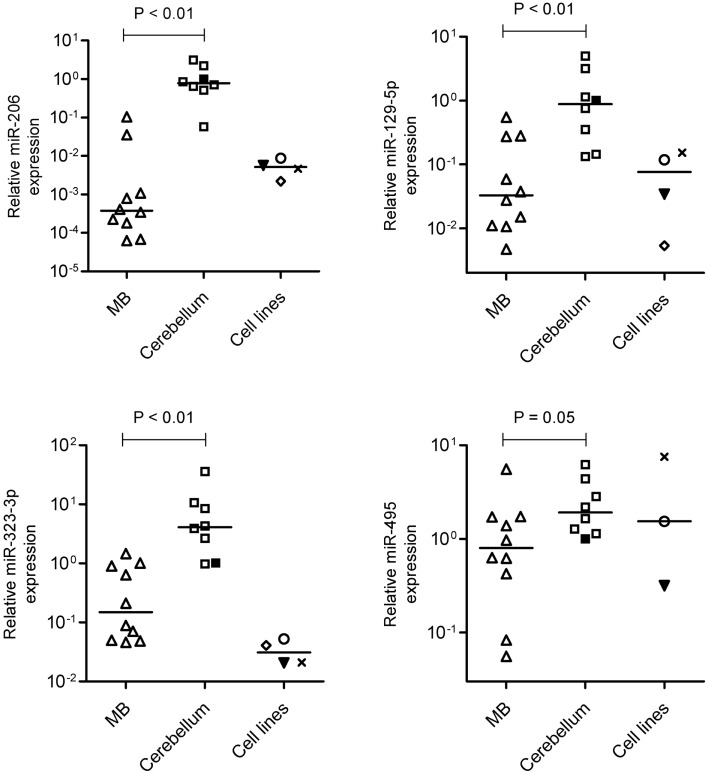
**Validation of miR-206, 129-5p, 323-3p, and 495 downregulation by reverse transcriptase-quantitative PCR**. Expression levels of miR-206, 129-5p, 323-3p, and 495 were performed in MB, normal fetal/newborn cerebellum and four different MB cell lines. Expression of miR-495 was not performed in D341 cell line. ○, DAOY; ▾, Meb-Med-8A; x, D283Med; ♢, D341. Comparisons of MB versus normal cerebellum were done by the Mann–Whitney test.

Real-time RT-qPCR analysis were performed with all samples used in the microarray analysis (*n* = 6 MB and *n* = 6 cerebellum) plus four other samples of MB (Table 1) and two new fetal/NW cerebellum controls (Table 2). As expected, miR-206 (*p* = 0.0001; Mann–Whitney test), miR-129-5p (*p* = 0.002), miR-323-3p (*p* = 0.014), and miR-495 (*p* = 0,054), had lower expression in MB in comparison to normal cerebellum (Figure 3), thus confirming our microarray findings. Expression of miR-206, 129-5p, 323-3p, and 495 were also investigated in a representative panel of MB cell lines. Compared with normal human cerebellum, miR-206, 129-5p, and 323-3p expression were found to be downregulated in all MB cell line tested (Figure 3).

### Upregulation of miR-206, 129-5p, and 323-3p in DAOY cells

**Figure 4:**
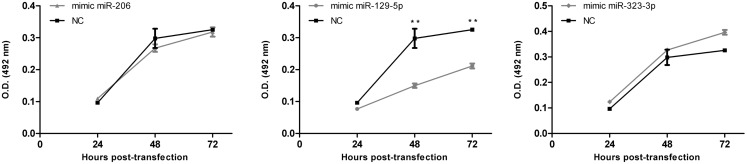
**Effect of miR-206, 129-5p, and 323-3p overexpression in DAOY cells proliferation**. DAOY cells were transiently transfected with miRNAs mimic and replated after 20 h at a 1,500 cells/well density. Cell proliferation was evaluated by the MTS assay at 4, 28, and 52 h after re-plating, thus after 24, 48, and 72 h post-transfection. Results are representative of three independent experiments. Mean ± SE are shown; **P < 0.01 according to two-tailed *t*-test; NC, negative control miRNA mimic (scrambled).

**Figure A2:**
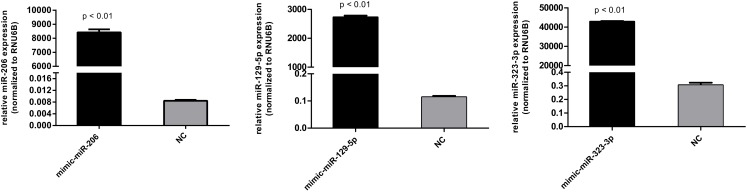
**Transfection efficiency of miR-206, miR-129-5p, and miR-323-3p in DAOY cells as evaluated by RT-qPCR**. Cells transfected with the scrambled mimic were included as negative control (NC). Values were normalized to the RUN6B endogenous control RNA. Relative expression values were calculated using the 2^−ΔΔ*Ct*^ method. Bars represent mean ± SD. Comparisons were performed by the Mann–Whitney test.

**Figure A3:**
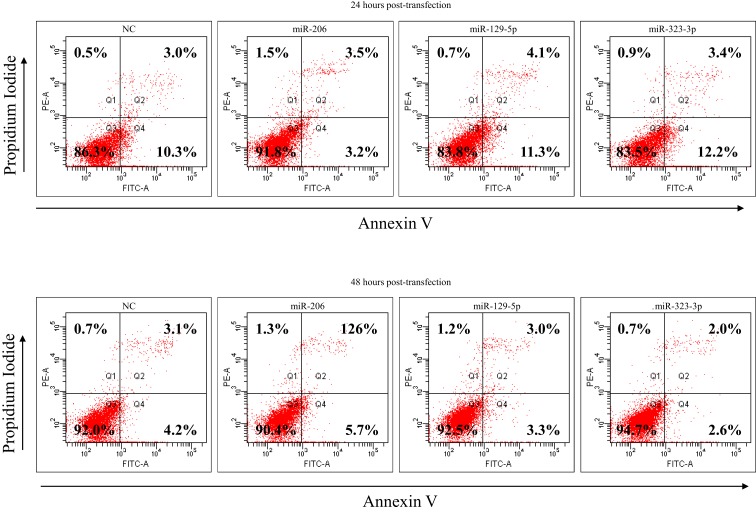
**Apoptosis analysis of DAOY cells at 24 and 48 h post-transfection with miR-206, 129-5p, 323-3p, or negative control mimics, as evaluated by Annexin V and propidium iodide staining and FACS analysis**. DAOY cells grown in serum-free RPMI-1640 medium. The percentage of necrotic (Q1), late apoptotic (Q2), viable (Q3), and early apoptotic (Q4) cells are shown in the corresponding quadrants. NC, negative control (scrambled mimic).

As a first approach to investigate the functional significance of miRNAs downregulation in MB, DAOY cells were transiently transfected with mimics of miR-206, 129-5p, 323-3p, or negative control #1. Transfection efficiency was confirmed by RT-qPCR (Figure A2 in Appendix). Twenty hours post-transfection, cells were collected and seeded in 96-well plates in serum-free medium. DAOY cells survive and even proliferate in serum-free medium for a short period of time. As shown in Figure 4, no consistent differences in proliferation were found in transfections with miR-206 and miR-323-3p. On the contrary, transfections with miR-129-5p resulted in a significant decrease in DAOY cell proliferation, as evaluated by the MTS assay (Figure 4). Similar experiments were conducted to evaluate cell survival and apoptosis by the Annexin V and propidium iodide staining methodology. No significant differences were found on cell viability or apoptosis after miR-206, miR-129-5p, and miR-323-3p transfections in comparison to control (Figure 4; Figure A3 in Appendix, respectively).

## Discussion

We investigated the expression profile of miRNAs in primary human MB of desmoplastic histology and SHH molecular subgroup, in comparison to normal fetal/newborn cerebellum. Eighty-four miRNAs were found to be differentially expressed in MB. The majority of these differentially expressed miRNAs were downregulated in comparison to normal cerebellum, corroborating previous studies. Most upregulated miRNAs identified in our study (12 out of 20) had been previously described in MB (12–14, 23, 28–32). On the contrary, 31 out of the 64 downregulated miRNAs are here described for the first time in association to MB (Table 3). Differences may be explained by the fact that a more comprehensive version of Affymetrix miRNA microarray was used in the present study. Moreover, previous studies included different subtypes of MB and a mix of children and adults cerebellum samples in their analysis (12, 13, 29). We believe that analysis on more uniform groups of both cancer and control samples may have helped us in detecting some smaller but consistent differences between groups.

A computational analysis was performed to predict the network and signaling pathways collectively targeted by the 64 downregulated and 20 upregulated miRNAs. Downregulated miRNAs in MB were predicted to target genes related to the ribosome, adherens junction, oxidative phosphorylation, metabolism of xenobiotics by cytochrome P450, and transforming growth factor-beta (*TGF-*β) signaling pathways. Axon guidance, *TGF-*β, *WNT*, insulin signaling pathways are known to play an important role in neurulation, CNS developmental, and/or MB pathogenesis (3, 33, 34). Since miRNA act as negative regulators of gene expression, a simpler interpretation of these findings is that MB has increased activation of these pathways in comparison to normal cerebellum.

Most importantly, half (32/64) of downregulated miRNAs reported in our study were found to belong to the cluster at 14q32 locus (also known as miR-154 cluster). This is in keeping with a previous study in a mouse model of MB, reporting that activation of SHH signaling leads to downregulation of the miR-154 cluster (35). Moreover, previous publications with primary MB found downregulation of some 14q32 miRNAs in MBs of the molecular subgroups WNT, SHH, and C as compared to normal cerebellum and MBs of subgroup D (13, 29). However, this is the first time that so many 14q32 miRNAs are shown to be downregulated in MB, thus suggesting a co-regulatory control of this cluster’s expression.

**Figure A4:**
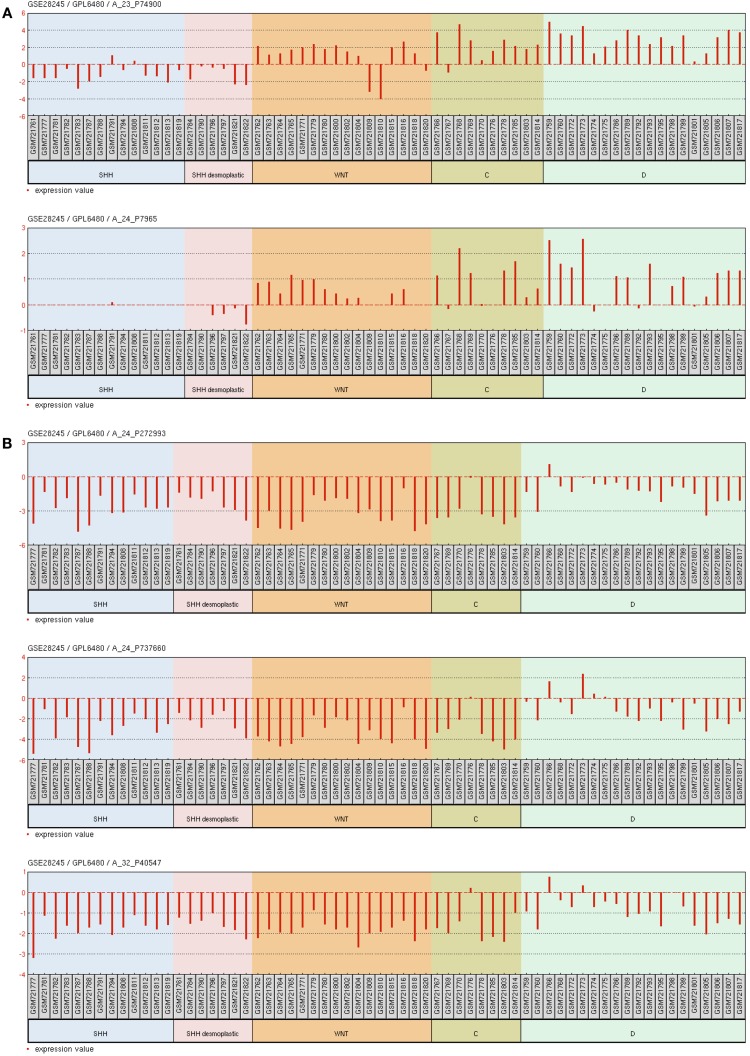
**Gene expression graph for different microarray probes of interest**. Data from 64 primary human MB samples accessible through GEO Series accession number GSE28245 (38) in NCBI’s Gene Expression Omnibus. **(A)** probe sets for *ESRRG*. **(B)** Probe sets for *MEG3.* Samples were grouped according to the molecular MB subgroups into WNT, SHH, C, and D. SHH desmoplastic was separated from SHH classic.

Deletions at locus 14q32 would be one possible explanation to the decreased 14q32 miRNAs expression. The recent analysis of somatic copy number aberrations in 1,087 MB samples report significant losses of chromosome arm 14q in the SHH subgroup of MBs, though not restricted to 14q32 (36). Alternative explanations are discussed below. Our Ingenuity pathways analysis pointed to nuclear orphan receptor *NR0B2* (also known as Small Heterodimer Partner, *SHP*) and (*ESRRG*) as possible controllers of 14q32 miRNA cluster expression. There is indeed experimental evidence in mouse showing that *NR0B2* is a repressor while *ESRRG* is and activator of a miRNA cluster in chromosome 12, which is ortholog to the 14q32 cluster in humans (37). Our analysis of microarray mRNA expression data for 64 primary human MB samples, accessible through GEO Series accession number GSE28245 (38) in NCBI’s Gene Expression Omnibus (39) revealed that *NR0B2* is not expressed in MB. Interestingly, *ESRRG* expression was found to be relatively high in MBs of the molecular subgroup D, intermediate in MBs of the WNT and C subgroups, and very low or absent in MBs of the SHH subgroup (Figure A4A in Appendix), thus reflecting 14q32 miRNAs abundance in each of the MB subgroup. These findings were confirmed by the analysis of gene expression data of an independent cohort of 90 primary MB samples (accession number GSE21166) deposited by Northcott et al. (13) (data not shown). *ESRRG* suppress cell proliferation in prostate cancer cells (40) and the estrogen receptor beta agonist diarylpropionitrile (*DPN*) exhibit a pro-apoptotic and anti-proliferative effect on MB (41). Experiments are warranted to investigate a possible causal connection between *ESRRG* and 14q32 miRNA cluster expression in MB.

The miRNA cluster at 14q32 lies within a parentally imprinted chromosomal area spanning genes *Dlk1*, *Meg3*, *Rtl1*, *Meg8*, and *Dio3* (42). *Dlk1*, *Rtl1*, and *Dio3* are paternally-, whereas Meg3 and Meg8 are maternally expressed transcripts (43). Imprinting of 14q32 is regulated, to some extent, by two intergenic differentially methylated regions known as IG-DMR and MEG3-DMR (44, 45). Deletions of the regulatory regions and/or epigenetic modifications may in theory cause aberrant 14q32 silencing in cancer. The recent 1,000 genome study of somatic copy number aberrations shows no recurrent focal deletions at locus 14q32 in MB (36). However, our analysis of public mRNA microarray expression data GSE28245 (38) revealed that *MEG3* is downregulated in MB in comparison to normal cerebellum. MBs of the molecular group C and WNT have the lowest expression, SHH has intermediate levels while group D have *MEG3* levels closer to normal cerebellum (Figure A4B in Appendix). Thus *MEG3* expression seems to correlate with the expression of 14q32 miRNAs among the different MB molecular groups, suggesting that the 14q32 miRNA locus may be under epigenetic regulation in MB. However, a genome wide analysis of promoter methylation on four primary MB samples showed no consistent methylation of 14q32 gene promoters (46). Although higher number of samples should be analyzed, this result corroborates findings in osteosarcoma, a tumor also presenting with downregulated 14q32 miRNAs expression and with no consistent changes in the methylation patterns at 14q32. Instead, silencing of 14q32 miRNA in osteosarcoma seems to be mediated by histone modification(s) (47).

Preliminary functional studies were performed in DAOY cells by ectopic expression of miR-129-5p, 206, and 323-3p mimics. Mimics for miR-206 and 323-3p had no significant effect on DAOY cells. miR-129-5p overexpression resulted in decreased cell proliferation, which may suggest a tumor suppressor role in MB.

## Conflict of Interest Statement

The authors declare that the research was conducted in the absence of any commercial or financial relationships that could be construed as a potential conflict of interest.

## Footnotes

^1^www.r-project.org

^2^www.metaboanalyst.ca

^3^http://diana.cslab.ece.ntua.gr/pathways/index_multiple.php

^4^http://www.ingenuity.com/
